# Crystal structure and Hirshfeld surface analysis of ethane-1,2-diaminium 3-[2-(1,3-dioxo-1,3-di­phenyl­propan-2-yl­idene)hydrazin­yl]-5-nitro-2-oxido­benzene­sulfonate dihydrate

**DOI:** 10.1107/S2056989018009118

**Published:** 2018-06-28

**Authors:** Zeliha Atioğlu, Mehmet Akkurt, Flavien A. A. Toze, Fatali E. Huseynov, Sarvinaz F. Hajiyeva

**Affiliations:** a İlke Education and Health Foundation, Cappadocia University, Cappadocia Vocational College, The Medical Imaging Techniques Program, 50420 Mustafapaşa, Ürgüp, Nevşehir, Turkey; b Department of Physics, Faculty of Sciences, Erciyes University, 38039 Kayseri, Turkey; c Department of Chemistry, Faculty of Sciences, University of Douala, PO Box 24157, Douala, Republic of , Cameroon; d Department of Ecology and Soil Sciences, Baku State University, Z. Xalilov Str. 23, Az 1148 Baku, Azerbaijan; e Organic Chemistry Department, Baku State University, Z. Xalilov Str. 23, Az 1148 Baku, Azerbaijan

**Keywords:** crystal structure, 5-nitro-2-oxido­benzene­sulfonate group, hydrogen bond, π–π stacking, Hirshfeld surface analysis

## Abstract

In the anion of the title hydrated salt, C_2_H_10_N_2_^2+^·C_21_H_13_N_3_O_8_S^2−^·2H_2_O, the planes of the phenyl rings and the benzene ring of the 5-nitro-2-oxido­benzene­sulfonate group are inclined to one another by 44.42 (11), 56.87 (11) and 77.70 (12)°. In the crystal, the anions are linked to the cations and the water mol­ecules by N—H⋯O and O—H⋯O hydrogen bonds, forming a three-dimensional network. Furthermore, there are face-to-face π–π stacking inter­actions between the centroids of one phenyl ring and the benzene ring of the 5-nitro-2-oxido­benzene­sulfonate group [centroid–centroid distance = 3.8382 (13) Å and slippage = 1.841 Å]. A Hirshfeld surface analysis was conducted to verify the contributions of the different inter­molecular inter­actions.

## Abstract 2

The title compound has a nonplanar conformation. In the crystal, the anions are linked to the cations and the water mol­ecules by N—H⋯O and O—H⋯O hydrogen bonds, forming a three-dimensional network. Face-to-face π–π stacking inter­actions are also observed.

## Chemical context   

Aryl­hydrazones of β-diketones (AHBD) and their complexes have attracted much attention due to their synthetic potential for organic and inorganic chemistries and diverse useful properties (Gurbanov *et al.*, 2017*a* ▸,*b* ▸; Jlassi *et al.*, 2014 ▸, 2018 ▸; Ma *et al.*, 2017*a* ▸,*b* ▸; Mahmudov & Pombeiro, 2016 ▸; Mahmudov *et al.*, 2014 ▸, 2017*a* ▸,*b* ▸). Usually, AHBDs have strong intra­molecular resonance-assisted hydrogen bonding (RAHB), which has a more profound effect on their reactivity (Mahmudov *et al.*, 2016 ▸) than regular hydrogen bonding and other types of noncovalent inter­actions (Ledenyova *et al.*, 2018 ▸; Mahmoudi *et al.*, 2016 ▸, 2018 ▸; Nasirova *et al.*, 2017 ▸; Politzer *et al.*, 2017 ▸; Scheiner, 2013 ▸; Shixaliyev *et al.*, 2018 ▸; Vandyshev *et al.*, 2017 ▸).
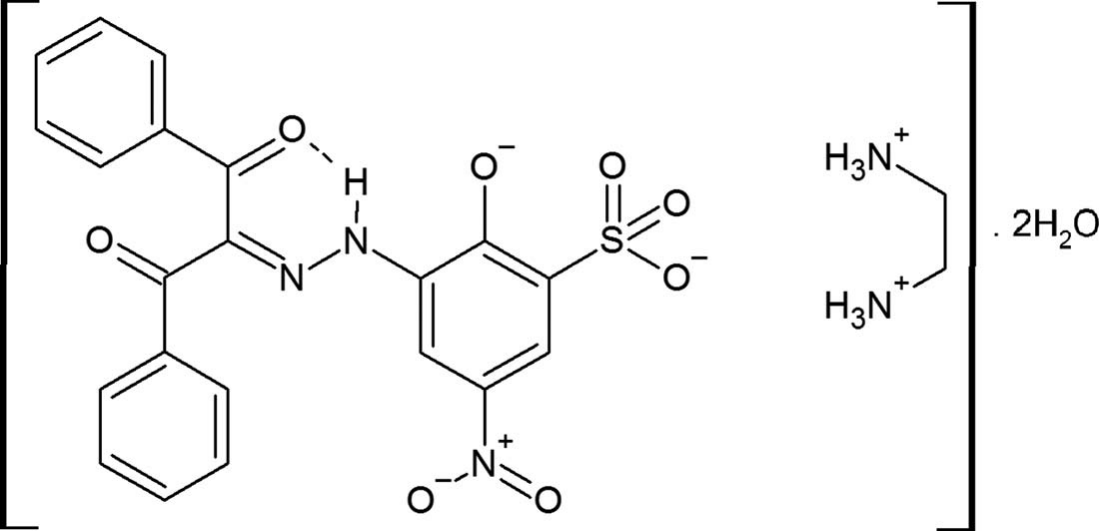


Herein we found the strong RAHB and inter­molecular charge-assisted hydrogen bonding that was expected in the title hydrated salt ethane-1,2-diaminium 3-[2-(1,3-dioxo-1,3-di­phenyl­propan-2-yl­idene)hydrazin­yl]-5-nitro-2-oxido­benzene­sulfonate dihydrate.

## Structural commentary   

**Figure 1:**
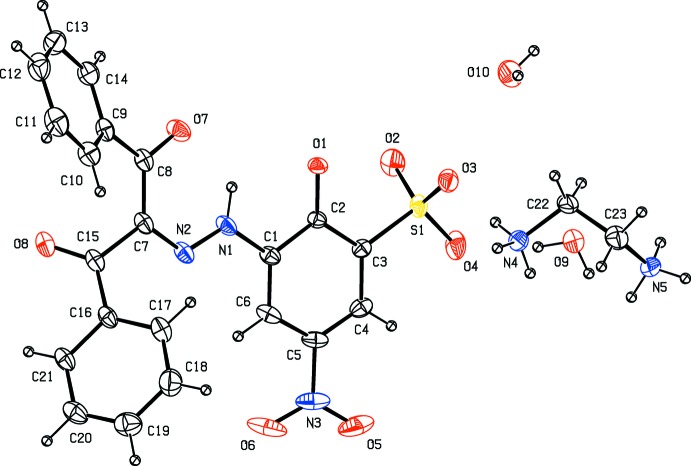
The mol­ecular structure of the title compound. Displacement ellipsoids are drawn at the 30% probability level. H atoms are shown as spheres of arbitrary radius.

In the anion of the title salt (Fig. 1 ▸), the planes of the phenyl rings (C9–C14 and C16–C21) and the benzene ring (C1–C6) of the 5-nitro-2-oxido­benzene­sulfonate group are inclined to one another by 44.42 (11), 56.87 (11) and 77.70 (12)°, respectively. The torsion angles O1—C2—C1—N1, C1—N1—N2—C7, N1—N2—C7—C8, N2—C7—C8—O7, N2—C7—C8—C9, N2—C7—C15—O8, N2—C7—C15—C16, C7—C15—C16—C17 and O8—C15—C16—C17 are 2.7 (3), −178.65 (19), −2.0 (3), −9.5 (3), 166.9 (2), 133.9 (2), −44.9 (3), −21.3 (3) and 159.9 (2)°, respectively. Therefore, the mol­ecular conformation of the title compound is not planar. The values of the geometric parameters of the title compound are within normal ranges (Allen *et al.*, 1987 ▸).

## Supra­molecular features and Hirshfeld surface analysis   

**Figure 2:**
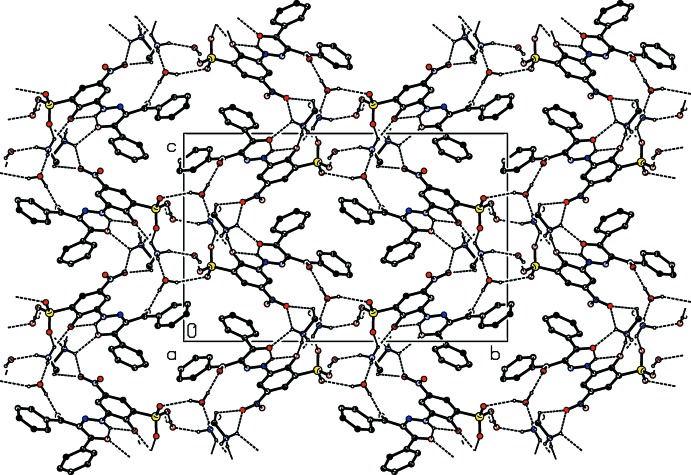
A view along the *a* axis of the packing and hydrogen bonding of the title compound.

In the crystal structure of the title compound, the anions are linked to the cations and two water mol­ecules by N—H⋯O and O—H⋯O hydrogen bonds, forming a three-dimensional network (Table 1 ▸ and Fig. 2 ▸). Furthermore, there are face-to-face π–π stacking inter­actions between the centroids of one phenyl ring (atoms C1–C6, *Cg*1) and the benzene ring of the 5-nitro-2-oxido­benzene­sulfonate group (*Cg*2) [*Cg*1⋯*Cg*2^a^ = 3.8382 (13) Å and slippage = 1.841 Å; symmetry code: (a) *x* + 1, −*y* + 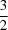, *z* + 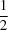].

**Figure 3:**
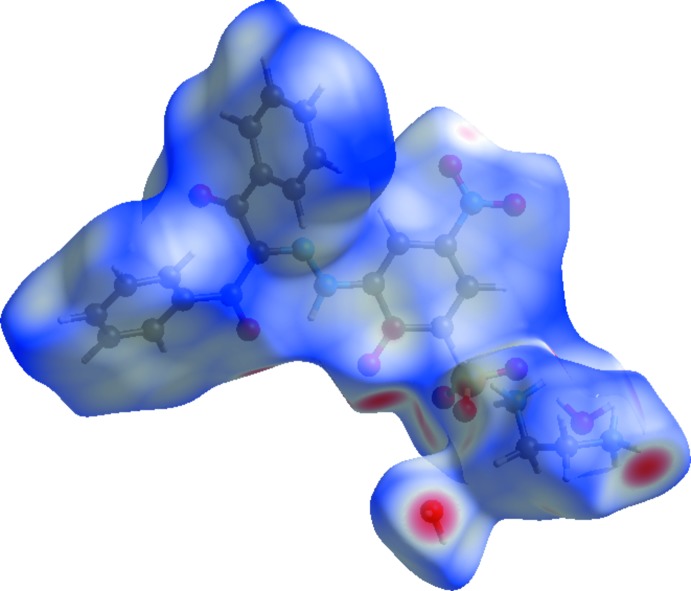
The Hirshfeld surface of the title compound mapped with *d*_norm_.

The Hirshfeld surface mapped over *d*_norm_ (McKinnon *et al.*, 2004 ▸; Spackman & Jayatilaka, 2009 ▸) for the title compound is depicted in Fig. 3 ▸. The red areas on the surface indicate short contacts as compared to the sum of the van der Waals radii, the blue areas indicate long contacts and the white areas indicate contacts with distances equal to the sum of the van der Waals radii. The highlighted red area shows the O—H⋯O hydrogen bonding, which is responsible for connecting anions and cations to each other.

**Figure 4:**
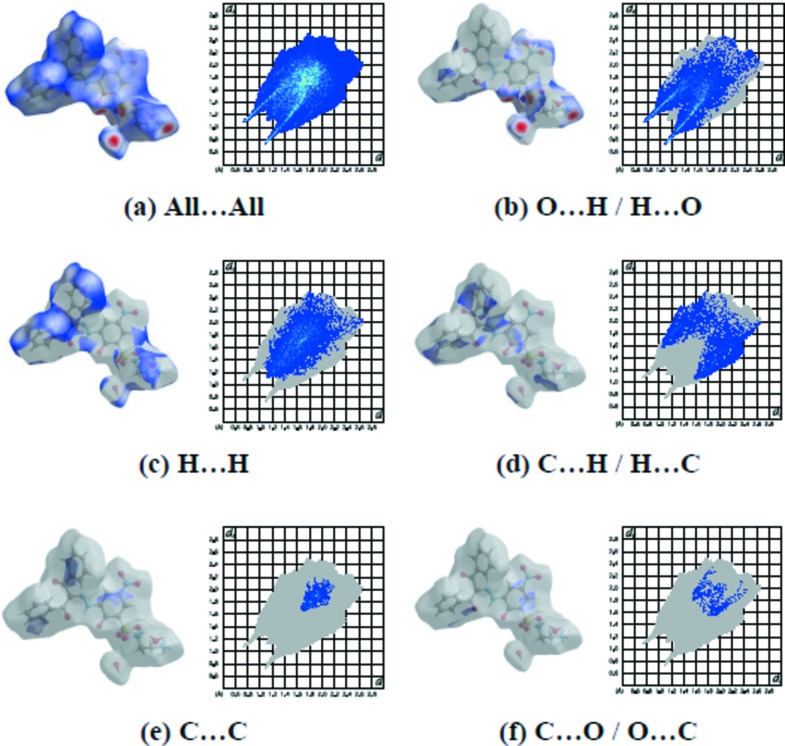
The two-dimensional fingerprint plots of the title compound, showing (*a*) all inter­actions, and delineated into (*b*) H⋯H, (*c*) O⋯H/H⋯O, (*d*) H⋯N/N⋯H, (*e*) C⋯O/O⋯C and (*f*) C⋯H/H⋯C inter­actions [*d*_e_ and *d*_i_ represent the distances from a point on the Hirshfeld surface to the nearest atoms outside (external) and inside (inter­nal) the surface, respectively].

**Figure 5:**
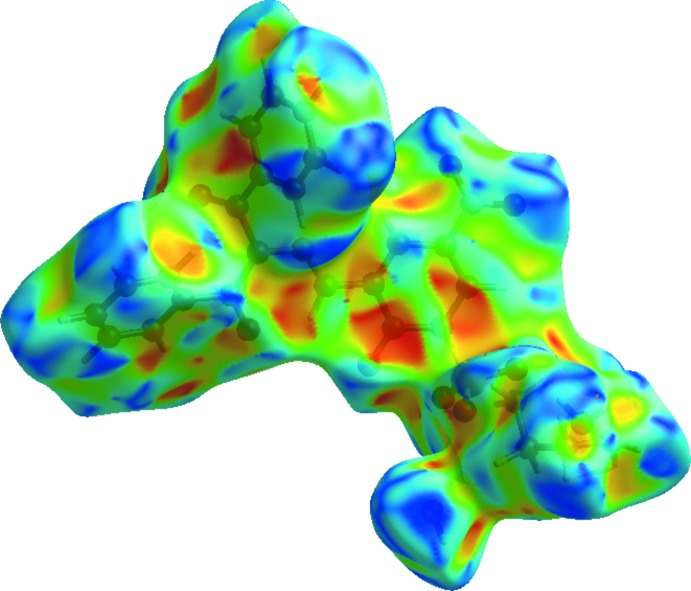
View of the three-dimensional Hirshfeld surface of the title complex plotted over shape index.

The overall two-dimensional fingerprint plot for the title compound and those delineated into O⋯H/H⋯O, H⋯H, C⋯H/H⋯C, C⋯C and C⋯O/O⋯C contacts are illustrated in Fig. 4 ▸; the percentage contributions from the different inter­atomic contacts to the Hirshfeld surfaces are as follows: O⋯H/H⋯O (39.5%), H⋯H (33.8%), C⋯H/H⋯C (14.5%), C⋯C (4.3%) and C⋯O/O⋯C (2.4%). The contributions of the other weak inter­molecular contacts to the Hirshfeld surfaces are listed in Table 2 ▸. The large number of O⋯H/H⋯O, H⋯H, C⋯H/H⋯C, C⋯C and C⋯O/O⋯C inter­actions suggest that van der Waals inter­actions and hydrogen bonding play the greatest roles in the crystal packing (Hathwar *et al.*, 2015 ▸). A view of the Hirshfeld surface of the title complex plotted over the shape index is given in Fig. 5 ▸.

## Synthesis and crystallization   

Synthesis of 3-[2-(1,3-dioxo-1,3-di­phenyl­propan-2-yl­idene)hydrazine­yl]-2-hy­droxy-5-nitrobenzene­sulfonic acid (H_3_ *L*) and its characterization by elemental analysis, ^1^H/^13^C NMR and IR was reported in Kuznik *et al.* (2011 ▸). 469 mg (1 mmol) of H_3_ *L* was dissolved in 30 ml of methanol and 0.06 ml (1 mmol) of ethyl­enedi­amine was added, with stirring for 5 min at room temperature (rt). The reaction mixture was then kept in air at rt for slow evaporation. After *ca* 2–3 d, orange crystals of the title compound were formed (yield 84%, based on H_3_ *L*). The final product was soluble in acetone, dimethyl sulfoxide (DMSO), ethanol and di­methyl­formamide (DMF), and insoluble in non-polar solvents. Elemental analysis for C_23_H_27_N_5_O_10_S, found (calculated) (%): C 48.79 (48.85), H 4.77 (4.81), N 12.27 (12.38). IR (KBr): 3470 ν(OH), 2989 ν(NH), 1667 ν(C=O), 1613 ν(C=O⋯H), 1576 ν(C=N) cm^−1^. ^1^H NMR (DMSO, inter­nal TMS): δ 3.86 (4H, 2CH_2_), 7.32–8.43 (12H, Ar—H), 10.13 (6H, 2NH_3_), 14.36 (*s*, 1H, N—H). ^13^C NMR (DMSO, inter­nal TMS): δ 41.18 (2CH_2_), 109.43 (2Ar—H), 123.01 (2Ar—H), 127.72 (2Ar—H), 128.28 (2Ar—H), 130.35 (Ar—H), 132.52 (Ar—H), 132.67 (Ar—H), 132.88 (Ar—H), 133.13 (Ar—H), 133.57 (Ar—CO), 133.80 (Ar—CO), 134.25 (C=N), 137.89 (Ar—SO_3_^−^), 143.38 (Ar—NH—N), 146.15 (Ar-NO_2_), 160.72 (Ar—O^−^), 191.37 (C=O), 191.89 (C=O).

## Refinement details   

Crystal data, data collection and structure refinement details are summarized in Table 3 ▸. All H atoms were placed in geometrically idealized positions and constrained to ride on their parent atoms, with O—H = 0.85 Å, N—H = 0.90 Å and C—H = 0.93–0.97 Å, and *U*_iso_(H) = 1.5*U*_eq_(O) and 1.2*U*_eq_(C,N).

## Supplementary Material

Crystal structure: contains datablock(s) I. DOI: 10.1107/S2056989018009118/qm2125sup1.cif

Structure factors: contains datablock(s) I. DOI: 10.1107/S2056989018009118/qm2125Isup2.hkl

Supporting information file. DOI: 10.1107/S2056989018009118/qm2125Isup3.cml

CCDC reference: 1851087

Additional supporting information:  crystallographic information; 3D view; checkCIF report

## Figures and Tables

**Table 1:** Hydrogen-bond geometry (Å, °)

*D*—H⋯*A*	*D*—H	H⋯*A*	*D*⋯*A*	*D*—H⋯*A*
O10—H10*A*⋯O8^i^	0.85	2.10	2.928 (2)	165
O9—H9*A*⋯O4	0.85	1.99	2.827 (2)	169
O9—H9*B*⋯O2^ii^	0.85	2.03	2.866 (2)	170
O10—H10*B*⋯O4^iii^	0.85	2.36	3.139 (3)	152
N1—H1*N*⋯O7	0.90	1.92	2.568 (2)	127
N4—H4*A*⋯O1^ii^	0.90	1.94	2.826 (2)	167
N4—H4*B*⋯O6^iv^	0.90	2.30	2.960 (2)	130
N4—H4*B*⋯O7^ii^	0.90	2.24	2.797 (2)	119
N5—H5*B*⋯O1^ii^	0.90	2.01	2.864 (2)	158
N4—H4*B*⋯O6^iv^	0.90	2.30	2.960 (2)	130
N4—H4*B*⋯O7^ii^	0.90	2.24	2.797 (2)	119
N4—H4*C*⋯O3	0.90	1.86	2.756 (2)	177
N5—H5*A*⋯O10^ii^	0.90	1.98	2.775 (3)	146
N5—H5*B*⋯O3^ii^	0.90	2.32	2.778 (2)	112
N5—H5*C*⋯O9^v^	0.90	1.98	2.835 (3)	159

Symmetry codes: (i) 
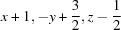
; (ii) 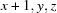; (iii) 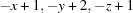; (iv) 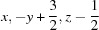; (v) 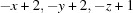.

**Table 2:** Percentage contributions of inter­atomic contacts to the Hirshfeld surface for the title compound

Contact	Percentage contribution
O⋯H/H⋯H	39.5
H⋯H	33.8
C⋯H/H⋯C	14.5
C⋯C	4.3
C⋯O/O⋯C	2.4
N⋯O/O⋯N	1.8
C⋯N/N⋯C	1.5
N⋯H/H⋯N	1.1
O⋯O	1.1

**Table 3:** Experimental details

Crystal data	
Chemical formula	C_2_H_10_N_2_^2+^·C_21_H_13_N_3_O_8_S^2−^·2H_2_O
*M*_r_	565.55
Crystal system, space group	Monoclinic, *P*2_1_/*c*
Temperature (K)	296
*a*, *b*, *c* (Å)	7.0590 (6), 23.851 (2), 15.3622 (13)
β (°)	93.337 (3)
*V* (Å^3^)	2582.1 (4)
*Z*	4
Radiation type	Mo *K*α
μ (mm^−1^)	0.19
Crystal size (mm)	0.26 × 0.15 × 0.08

Data collection	
Diffractometer	Bruker APEXII CCD
Absorption correction	Multi-scan (*SADABS*; Bruker, 2007 ▸)
*T*_min_, *T*_max_	0.946, 0.975
No. of measured, independent and observed [*I* > 2σ(*I*)] reflections	41494, 4930, 3559
*R*_int_	0.083
(sin θ/λ)_max_ (Å^−1^)	0.611

Refinement	
*R*[*F* ^2^ > 2σ(*F* ^2^)], *wR*(*F* ^2^), *S*	0.043, 0.112, 1.02
No. of reflections	4930
No. of parameters	352
H-atom treatment	H-atom parameters constrained
Δρ_max_, Δρ_min_ (e Å^−3^)	0.37, −0.34

Computer programs: *APEX2* and *SAINT* (Bruker, 2007 ▸), *SHELXS97* (Sheldrick, 2008 ▸), *SHELXL2018* (Sheldrick, 2015 ▸), *ORTEP-3 for Windows* (Farrugia, 2012 ▸) and *PLATON* (Spek, 2009 ▸).

## supplementary crystallographic information

### Crystal data

**Table:**

C_2_H_10_N_2_^2+^·C_21_H_13_N_3_O_8_S^2^^−^·2H_2_O	*F*(000) = 1184
*M**_r_* = 565.55	*D*_x_ = 1.455 Mg m^−^^3^
Monoclinic, *P*2_1_/*c*	Mo *K*α radiation, λ = 0.71073 Å
*a* = 7.0590 (6) Å	Cell parameters from 7684 reflections
*b* = 23.851 (2) Å	θ = 2.7–25.0°
*c* = 15.3622 (13) Å	µ = 0.19 mm^−^^1^
β = 93.337 (3)°	*T* = 296 K
*V* = 2582.1 (4) Å^3^	Plate, orange
*Z* = 4	0.26 × 0.15 × 0.08 mm

### Data collection

**Table:**

Bruker APEXII CCD diffractometer	3559 reflections with *I* > 2σ(*I*)
φ and ω scans	*R*_int_ = 0.083
Absorption correction: multi-scan (SADABS; Bruker, 2007)	θ_max_ = 25.8°, θ_min_ = 2.7°
*T*_min_ = 0.946, *T*_max_ = 0.975	*h* = −8→8
41494 measured reflections	*k* = −29→29
4930 independent reflections	*l* = −18→17

### Refinement

**Table:**

Refinement on *F*^2^	0 restraints
Least-squares matrix: full	Hydrogen site location: mixed
*R*[*F*^2^ > 2σ(*F*^2^)] = 0.043	H-atom parameters constrained
*wR*(*F*^2^) = 0.112	*w* = 1/[σ^2^(*F*_o_^2^) + (0.053*P*)^2^ + 0.9465*P*] where *P* = (*F*_o_^2^ + 2*F*_c_^2^)/3
*S* = 1.02	(Δ/σ)_max_ = 0.001
4930 reflections	Δρ_max_ = 0.37 e Å^−^^3^
352 parameters	Δρ_min_ = −0.34 e Å^−^^3^

### Special details

**Table:**

Geometry. All esds (except the esd in the dihedral angle between two l.s. planes) are estimated using the full covariance matrix. The cell esds are taken into account individually in the estimation of esds in distances, angles and torsion angles; correlations between esds in cell parameters are only used when they are defined by crystal symmetry. An approximate (isotropic) treatment of cell esds is used for estimating esds involving l.s. planes.

### Fractional atomic coordinates and isotropic or equivalent isotropic displacement parameters (Å^2^)

**Table:**

	*x*	*y*	*z*	*U*_iso_*/*U*_eq_	
C1	0.1741 (3)	0.76729 (8)	0.65426 (13)	0.0333 (5)	
C2	0.2217 (3)	0.82248 (8)	0.62626 (12)	0.0288 (4)	
C3	0.3962 (3)	0.84445 (8)	0.66401 (12)	0.0299 (4)	
C4	0.5152 (3)	0.81269 (10)	0.71878 (13)	0.0375 (5)	
H4	0.630239	0.827277	0.740985	0.045*	
C5	0.4612 (3)	0.75882 (10)	0.74031 (14)	0.0411 (5)	
C6	0.2903 (3)	0.73575 (9)	0.70886 (14)	0.0408 (5)	
H6	0.255832	0.699667	0.724575	0.049*	
C7	−0.2184 (3)	0.67860 (9)	0.59205 (13)	0.0364 (5)	
C8	−0.3471 (3)	0.71352 (9)	0.53687 (14)	0.0374 (5)	
C9	−0.5092 (3)	0.68929 (9)	0.48251 (13)	0.0369 (5)	
C10	−0.5022 (3)	0.63658 (10)	0.44507 (15)	0.0451 (6)	
H10	−0.397420	0.613599	0.456841	0.054*	
C11	−0.6522 (4)	0.61810 (11)	0.38988 (17)	0.0572 (7)	
H11	−0.646209	0.583020	0.363766	0.069*	
C12	−0.8086 (4)	0.65112 (12)	0.37366 (17)	0.0595 (7)	
H12	−0.909617	0.638138	0.337620	0.071*	
C13	−0.8166 (4)	0.70340 (12)	0.41054 (17)	0.0566 (7)	
H13	−0.923465	0.725723	0.399761	0.068*	
C14	−0.6665 (3)	0.72290 (10)	0.46357 (15)	0.0450 (6)	
H14	−0.670797	0.758854	0.486795	0.054*	
C15	−0.2678 (3)	0.62089 (9)	0.62050 (13)	0.0379 (5)	
C16	−0.1207 (3)	0.57670 (8)	0.62333 (13)	0.0365 (5)	
C17	0.0414 (3)	0.57992 (10)	0.57562 (16)	0.0489 (6)	
H17	0.060741	0.611197	0.541097	0.059*	
C18	0.1726 (4)	0.53723 (11)	0.57924 (19)	0.0602 (7)	
H18	0.278000	0.539226	0.545767	0.072*	
C19	0.1484 (4)	0.49156 (11)	0.63225 (18)	0.0587 (7)	
H19	0.239317	0.463295	0.635947	0.070*	
C20	−0.0099 (4)	0.48781 (10)	0.67960 (16)	0.0499 (6)	
H20	−0.025764	0.456967	0.715476	0.060*	
C21	−0.1450 (3)	0.52920 (9)	0.67446 (14)	0.0414 (5)	
H21	−0.253732	0.525567	0.705362	0.050*	
C22	0.8316 (3)	0.90536 (10)	0.39177 (16)	0.0473 (6)	
H22A	0.824565	0.944179	0.410147	0.057*	
H22B	0.740222	0.900097	0.342811	0.057*	
C23	1.0255 (3)	0.89378 (11)	0.36299 (15)	0.0492 (6)	
H23A	1.044320	0.853556	0.359854	0.059*	
H23B	1.036856	0.909075	0.305023	0.059*	
N1	−0.0004 (3)	0.74786 (7)	0.61817 (11)	0.0383 (4)	
H1N	−0.064888	0.772475	0.583317	0.046*	
N2	−0.0546 (3)	0.69610 (7)	0.62829 (11)	0.0385 (4)	
N3	0.5811 (4)	0.72606 (10)	0.79974 (13)	0.0586 (6)	
N4	0.7806 (2)	0.86879 (8)	0.46401 (12)	0.0400 (4)	
H4A	0.877010	0.865031	0.504591	0.048*	
H4B	0.740870	0.835741	0.441351	0.048*	
H4C	0.684390	0.883711	0.492181	0.048*	
N5	1.1737 (2)	0.91827 (8)	0.42271 (12)	0.0439 (5)	
H5A	1.285858	0.920295	0.397881	0.053*	
H5B	1.185958	0.899605	0.473641	0.053*	
H5C	1.149575	0.954525	0.433951	0.053*	
O1	0.11362 (19)	0.84871 (6)	0.57058 (9)	0.0358 (3)	
O2	0.3021 (2)	0.94939 (7)	0.66294 (12)	0.0558 (5)	
O3	0.4796 (2)	0.91527 (7)	0.54483 (10)	0.0464 (4)	
O4	0.6324 (2)	0.92621 (8)	0.68812 (11)	0.0623 (5)	
O5	0.7429 (3)	0.74247 (10)	0.81985 (14)	0.0839 (7)	
O6	0.5162 (4)	0.68241 (9)	0.83004 (13)	0.0827 (7)	
O7	−0.3171 (2)	0.76438 (6)	0.53110 (11)	0.0495 (4)	
O8	−0.4290 (2)	0.61161 (7)	0.64236 (12)	0.0555 (5)	
O9	0.9258 (2)	0.96664 (7)	0.58733 (11)	0.0505 (4)	
H9A	0.846428	0.950189	0.618111	0.076*	
H9B	1.031219	0.959099	0.614351	0.076*	
O10	0.4185 (3)	0.94367 (8)	0.29341 (12)	0.0612 (5)	
H10A	0.441072	0.925449	0.247559	0.092*	
H10B	0.432662	0.978519	0.284149	0.092*	
S1	0.45667 (7)	0.91402 (2)	0.63830 (3)	0.03736 (16)	

### Atomic displacement parameters (Å^2^)

**Table:**

	*U*^11^	*U*^22^	*U*^33^	*U*^12^	*U*^13^	*U*^23^
C1	0.0437 (12)	0.0288 (11)	0.0265 (10)	−0.0023 (9)	−0.0048 (9)	0.0007 (8)
C2	0.0316 (10)	0.0282 (10)	0.0261 (10)	0.0006 (8)	−0.0018 (8)	0.0003 (8)
C3	0.0301 (10)	0.0344 (11)	0.0249 (10)	0.0002 (8)	−0.0014 (8)	−0.0032 (8)
C4	0.0336 (11)	0.0518 (14)	0.0264 (11)	0.0056 (10)	−0.0044 (8)	−0.0058 (10)
C5	0.0521 (13)	0.0422 (13)	0.0278 (11)	0.0167 (11)	−0.0074 (10)	0.0016 (9)
C6	0.0630 (15)	0.0269 (11)	0.0314 (11)	0.0034 (10)	−0.0058 (10)	0.0037 (9)
C7	0.0476 (13)	0.0301 (11)	0.0315 (11)	−0.0094 (9)	0.0016 (9)	0.0021 (9)
C8	0.0470 (12)	0.0313 (12)	0.0343 (11)	−0.0081 (9)	0.0062 (9)	0.0000 (9)
C9	0.0461 (12)	0.0358 (12)	0.0292 (11)	−0.0096 (10)	0.0061 (9)	−0.0004 (9)
C10	0.0550 (14)	0.0399 (13)	0.0403 (13)	−0.0071 (11)	0.0027 (11)	−0.0053 (10)
C11	0.0754 (19)	0.0482 (15)	0.0479 (15)	−0.0201 (14)	0.0010 (13)	−0.0089 (12)
C12	0.0593 (17)	0.0687 (19)	0.0493 (16)	−0.0245 (15)	−0.0060 (13)	0.0022 (13)
C13	0.0438 (14)	0.0718 (19)	0.0539 (16)	−0.0033 (13)	0.0015 (12)	0.0090 (14)
C14	0.0511 (14)	0.0435 (13)	0.0409 (13)	−0.0033 (11)	0.0062 (11)	−0.0002 (10)
C15	0.0503 (13)	0.0337 (12)	0.0297 (11)	−0.0117 (10)	0.0021 (9)	0.0032 (9)
C16	0.0492 (12)	0.0280 (11)	0.0319 (11)	−0.0118 (9)	−0.0014 (9)	0.0005 (9)
C17	0.0588 (15)	0.0391 (13)	0.0495 (14)	−0.0103 (11)	0.0094 (12)	0.0042 (11)
C18	0.0558 (16)	0.0540 (17)	0.0721 (19)	−0.0030 (13)	0.0160 (14)	−0.0007 (14)
C19	0.0655 (17)	0.0432 (15)	0.0666 (18)	0.0051 (13)	−0.0044 (14)	−0.0056 (13)
C20	0.0708 (17)	0.0320 (13)	0.0455 (14)	−0.0066 (12)	−0.0066 (12)	0.0025 (10)
C21	0.0564 (14)	0.0310 (12)	0.0366 (12)	−0.0124 (10)	0.0018 (10)	0.0020 (9)
C22	0.0426 (13)	0.0460 (14)	0.0513 (14)	−0.0101 (10)	−0.0159 (11)	0.0175 (11)
C23	0.0549 (15)	0.0589 (15)	0.0330 (12)	−0.0100 (12)	−0.0057 (10)	0.0049 (11)
N1	0.0505 (11)	0.0254 (9)	0.0376 (10)	−0.0106 (8)	−0.0093 (8)	0.0062 (7)
N2	0.0524 (11)	0.0290 (9)	0.0339 (10)	−0.0095 (8)	−0.0003 (8)	0.0043 (8)
N3	0.0747 (16)	0.0610 (15)	0.0377 (12)	0.0342 (13)	−0.0185 (11)	−0.0060 (11)
N4	0.0350 (9)	0.0419 (11)	0.0417 (10)	−0.0091 (8)	−0.0088 (8)	0.0025 (8)
N5	0.0325 (9)	0.0538 (12)	0.0448 (11)	−0.0005 (8)	−0.0036 (8)	0.0119 (9)
O1	0.0338 (7)	0.0317 (8)	0.0402 (8)	−0.0044 (6)	−0.0130 (6)	0.0089 (6)
O2	0.0625 (11)	0.0325 (9)	0.0735 (12)	−0.0020 (8)	0.0133 (9)	−0.0145 (8)
O3	0.0416 (9)	0.0621 (11)	0.0353 (9)	−0.0095 (8)	−0.0007 (7)	0.0085 (7)
O4	0.0527 (10)	0.0841 (14)	0.0479 (10)	−0.0380 (9)	−0.0151 (8)	0.0087 (9)
O5	0.0624 (13)	0.1168 (19)	0.0691 (14)	0.0341 (13)	−0.0251 (11)	0.0068 (13)
O6	0.132 (2)	0.0472 (12)	0.0640 (13)	0.0276 (12)	−0.0364 (13)	0.0117 (10)
O7	0.0617 (10)	0.0296 (9)	0.0552 (10)	−0.0094 (7)	−0.0123 (8)	0.0046 (7)
O8	0.0529 (11)	0.0488 (10)	0.0659 (12)	−0.0097 (8)	0.0134 (9)	0.0171 (9)
O9	0.0475 (9)	0.0469 (10)	0.0573 (10)	−0.0050 (7)	0.0047 (8)	0.0065 (8)
O10	0.0683 (12)	0.0507 (11)	0.0672 (12)	0.0072 (9)	0.0248 (9)	0.0019 (9)
S1	0.0352 (3)	0.0414 (3)	0.0351 (3)	−0.0143 (2)	−0.0016 (2)	−0.0014 (2)

### Geometric parameters (Å, º)

**Table:**

C1—C6	1.365 (3)	C17—H17	0.9300
C1—N1	1.400 (3)	C18—C19	1.377 (4)
C1—C2	1.431 (3)	C18—H18	0.9300
C2—O1	1.276 (2)	C19—C20	1.371 (4)
C2—C3	1.430 (3)	C19—H19	0.9300
C3—C4	1.380 (3)	C20—C21	1.372 (3)
C3—S1	1.764 (2)	C20—H20	0.9300
C4—C5	1.386 (3)	C21—H21	0.9300
C4—H4	0.9300	C22—N4	1.473 (3)
C5—C6	1.387 (3)	C22—C23	1.489 (3)
C5—N3	1.439 (3)	C22—H22A	0.9700
C6—H6	0.9300	C22—H22B	0.9700
C7—N2	1.321 (3)	C23—N5	1.472 (3)
C7—C8	1.466 (3)	C23—H23A	0.9700
C7—C15	1.492 (3)	C23—H23B	0.9700
C8—O7	1.236 (2)	N1—N2	1.305 (2)
C8—C9	1.493 (3)	N1—H1N	0.8999
C9—C10	1.385 (3)	N3—O5	1.230 (3)
C9—C14	1.387 (3)	N3—O6	1.239 (3)
C10—C11	1.389 (3)	N4—H4A	0.9000
C10—H10	0.9300	N4—H4B	0.9000
C11—C12	1.367 (4)	N4—H4C	0.8999
C11—H11	0.9300	N5—H5A	0.8999
C12—C13	1.372 (4)	N5—H5B	0.9000
C12—H12	0.9300	N5—H5C	0.8999
C13—C14	1.379 (4)	O2—S1	1.4468 (17)
C13—H13	0.9300	O3—S1	1.4546 (16)
C14—H14	0.9300	O4—S1	1.4484 (16)
C15—O8	1.225 (3)	O9—H9A	0.8500
C15—C16	1.479 (3)	O9—H9B	0.8498
C16—C21	1.395 (3)	O10—H10A	0.8505
C16—C17	1.396 (3)	O10—H10B	0.8502
C17—C18	1.376 (4)		
			
C6—C1—N1	123.00 (19)	C17—C18—C19	120.2 (3)
C6—C1—C2	123.24 (19)	C17—C18—H18	119.9
N1—C1—C2	113.73 (17)	C19—C18—H18	119.9
O1—C2—C3	124.02 (18)	C20—C19—C18	119.9 (3)
O1—C2—C1	120.68 (17)	C20—C19—H19	120.1
C3—C2—C1	115.29 (17)	C18—C19—H19	120.1
C4—C3—C2	121.70 (19)	C19—C20—C21	120.5 (2)
C4—C3—S1	120.40 (16)	C19—C20—H20	119.7
C2—C3—S1	117.90 (14)	C21—C20—H20	119.7
C3—C4—C5	119.2 (2)	C20—C21—C16	120.6 (2)
C3—C4—H4	120.4	C20—C21—H21	119.7
C5—C4—H4	120.4	C16—C21—H21	119.7
C4—C5—C6	122.05 (19)	N4—C22—C23	112.52 (19)
C4—C5—N3	119.7 (2)	N4—C22—H22A	109.1
C6—C5—N3	118.2 (2)	C23—C22—H22A	109.1
C1—C6—C5	118.4 (2)	N4—C22—H22B	109.1
C1—C6—H6	120.8	C23—C22—H22B	109.1
C5—C6—H6	120.8	H22A—C22—H22B	107.8
N2—C7—C8	124.18 (19)	N5—C23—C22	111.9 (2)
N2—C7—C15	112.46 (19)	N5—C23—H23A	109.2
C8—C7—C15	123.10 (19)	C22—C23—H23A	109.2
O7—C8—C7	119.8 (2)	N5—C23—H23B	109.2
O7—C8—C9	117.9 (2)	C22—C23—H23B	109.2
C7—C8—C9	122.19 (19)	H23A—C23—H23B	107.9
C10—C9—C14	119.0 (2)	N2—N1—C1	121.57 (18)
C10—C9—C8	122.6 (2)	N2—N1—H1N	123.1
C14—C9—C8	118.2 (2)	C1—N1—H1N	115.0
C9—C10—C11	119.8 (2)	N1—N2—C7	120.29 (18)
C9—C10—H10	120.1	O5—N3—O6	122.1 (2)
C11—C10—H10	120.1	O5—N3—C5	119.4 (3)
C12—C11—C10	120.5 (3)	O6—N3—C5	118.5 (2)
C12—C11—H11	119.8	C22—N4—H4A	111.9
C10—C11—H11	119.8	C22—N4—H4B	108.2
C11—C12—C13	120.0 (2)	H4A—N4—H4B	112.8
C11—C12—H12	120.0	C22—N4—H4C	110.6
C13—C12—H12	120.0	H4A—N4—H4C	105.5
C12—C13—C14	120.1 (3)	H4B—N4—H4C	107.8
C12—C13—H13	120.0	C23—N5—H5A	111.6
C14—C13—H13	120.0	C23—N5—H5B	112.0
C13—C14—C9	120.5 (2)	H5A—N5—H5B	110.6
C13—C14—H14	119.8	C23—N5—H5C	111.4
C9—C14—H14	119.8	H5A—N5—H5C	102.2
O8—C15—C16	121.69 (19)	H5B—N5—H5C	108.6
O8—C15—C7	118.9 (2)	H9A—O9—H9B	102.6
C16—C15—C7	119.36 (19)	H10A—O10—H10B	109.4
C21—C16—C17	118.1 (2)	O2—S1—O4	112.35 (11)
C21—C16—C15	119.1 (2)	O2—S1—O3	112.05 (11)
C17—C16—C15	122.75 (19)	O4—S1—O3	112.11 (10)
C18—C17—C16	120.6 (2)	O2—S1—C3	107.09 (10)
C18—C17—H17	119.7	O4—S1—C3	106.34 (10)
C16—C17—H17	119.7	O3—S1—C3	106.41 (9)
			
C6—C1—C2—O1	175.3 (2)	N2—C7—C15—O8	133.9 (2)
N1—C1—C2—O1	−2.7 (3)	C8—C7—C15—O8	−40.5 (3)
C6—C1—C2—C3	−3.9 (3)	N2—C7—C15—C16	−44.9 (3)
N1—C1—C2—C3	178.05 (17)	C8—C7—C15—C16	140.7 (2)
O1—C2—C3—C4	−174.87 (19)	O8—C15—C16—C21	−19.0 (3)
C1—C2—C3—C4	4.3 (3)	C7—C15—C16—C21	159.75 (19)
O1—C2—C3—S1	5.0 (3)	O8—C15—C16—C17	159.9 (2)
C1—C2—C3—S1	−175.82 (14)	C7—C15—C16—C17	−21.3 (3)
C2—C3—C4—C5	−2.4 (3)	C21—C16—C17—C18	0.1 (3)
S1—C3—C4—C5	177.78 (16)	C15—C16—C17—C18	−178.8 (2)
C3—C4—C5—C6	−0.3 (3)	C16—C17—C18—C19	−2.1 (4)
C3—C4—C5—N3	−177.87 (19)	C17—C18—C19—C20	2.0 (4)
N1—C1—C6—C5	179.35 (19)	C18—C19—C20—C21	0.2 (4)
C2—C1—C6—C5	1.5 (3)	C19—C20—C21—C16	−2.2 (4)
C4—C5—C6—C1	0.8 (3)	C17—C16—C21—C20	2.0 (3)
N3—C5—C6—C1	178.33 (19)	C15—C16—C21—C20	−179.0 (2)
N2—C7—C8—O7	−9.5 (3)	N4—C22—C23—N5	−77.7 (3)
C15—C7—C8—O7	164.2 (2)	C6—C1—N1—N2	−6.5 (3)
N2—C7—C8—C9	166.9 (2)	C2—C1—N1—N2	171.52 (18)
C15—C7—C8—C9	−19.4 (3)	C1—N1—N2—C7	−178.65 (19)
O7—C8—C9—C10	143.6 (2)	C8—C7—N2—N1	2.0 (3)
C7—C8—C9—C10	−32.9 (3)	C15—C7—N2—N1	−172.29 (18)
O7—C8—C9—C14	−31.3 (3)	C4—C5—N3—O5	−11.5 (3)
C7—C8—C9—C14	152.3 (2)	C6—C5—N3—O5	170.9 (2)
C14—C9—C10—C11	−0.4 (3)	C4—C5—N3—O6	167.5 (2)
C8—C9—C10—C11	−175.2 (2)	C6—C5—N3—O6	−10.2 (3)
C9—C10—C11—C12	−1.4 (4)	C4—C3—S1—O2	−123.34 (17)
C10—C11—C12—C13	1.4 (4)	C2—C3—S1—O2	56.81 (18)
C11—C12—C13—C14	0.4 (4)	C4—C3—S1—O4	−3.0 (2)
C12—C13—C14—C9	−2.2 (4)	C2—C3—S1—O4	177.11 (16)
C10—C9—C14—C13	2.1 (3)	C4—C3—S1—O3	116.66 (17)
C8—C9—C14—C13	177.2 (2)	C2—C3—S1—O3	−63.19 (17)

### Hydrogen-bond geometry (Å, º)

**Table:**

*D*—H···*A*	*D*—H	H···*A*	*D*···*A*	*D*—H···*A*
O10—H10*A*···O8^i^	0.85	2.10	2.928 (2)	165
O9—H9*A*···O4	0.85	1.99	2.827 (2)	169
O9—H9*B*···O2^ii^	0.85	2.03	2.866 (2)	170
O10—H10*B*···O4^iii^	0.85	2.36	3.139 (3)	152
N1—H1*N*···O7	0.90	1.92	2.568 (2)	127
N4—H4*A*···O1^ii^	0.90	1.94	2.826 (2)	167
N4—H4*B*···O6^iv^	0.90	2.30	2.960 (2)	130
N4—H4*B*···O7^ii^	0.90	2.24	2.797 (2)	119
N5—H5*B*···O1^ii^	0.90	2.01	2.864 (2)	158
N4—H4*B*···O6^iv^	0.90	2.30	2.960 (2)	130
N4—H4*B*···O7^ii^	0.90	2.24	2.797 (2)	119
N4—H4*C*···O3	0.90	1.86	2.756 (2)	177
N5—H5*A*···O10^ii^	0.90	1.98	2.775 (3)	146
N5—H5*B*···O3^ii^	0.90	2.32	2.778 (2)	112
N5—H5*C*···O9^v^	0.90	1.98	2.835 (3)	159

Symmetry codes: (i) *x*+1, −*y*+3/2, *z*−1/2; (ii) *x*+1, *y*, *z*; (iii) −*x*+1, −*y*+2, −*z*+1; (iv) *x*, −*y*+3/2, *z*−1/2; (v) −*x*+2, −*y*+2, −*z*+1.
